# Human Papillomavirus Detection and Abnormal Anal Cytology in HIV-infected Patients Using p16/Ki-67 Dual-Staining

**DOI:** 10.31557/APJCP.2020.21.7.2013

**Published:** 2020-07

**Authors:** Natcha Patarapadungkit, Parinyabhorn Khonhan, Pornrith Pisuttimarn, Chamsai Pientong, Tipaya Ekalaksananan, Supinda Koonmee

**Affiliations:** 1 *Department of Pathology, Faculty of Medicine, Khon Kaen University, Khon Kaen, Thailand. *; 2 *HPV & EBV and carcinogenesis Research Group, Khon Kaen University, Khon Kaen, Thailand. *; 3 *Sirindhorn Hospital, Khon Kaen, Thailand. *; 4 *Department of Microbiology, Faculty of Medicine, Khon Kaen University, Khon Kaen, Thailand. *

**Keywords:** Anal cytology, HIV, HPV, immunocytochemistry, p16/ki67, dual-staining

## Abstract

**Objective:**

We investigated human papillomavirus (HPV) infection and detected anal squamous intraepithelial lesions by modified liquid-based cytology (LBC) and p16/Ki67 dual-staining.

**Methods:**

Anal swabs (n=393) were collected from patients with HIV infection. Anal cells were kept in 95% ethyl alcohol for modified LBC. DNA was extracted from cells for HPV detection and genotyping using real-time PCR and reverse line blot hybridization. Results: Nine samples (2.3%) were unsatisfactory specimens, 74.8% (294/393) were negative for intraepithelial malignancies (NILM) and 22.9% (90/393) exhibited squamous intraepithelial lesions (SIL). In the latter category, 13.7% of samples (54/393) contained atypical squamous cells of undetermined significance (ASCUS), 6.9% (27/393) were classified as low-grade SIL (LSIL) and 2.3% (9/393) as high-grade SIL (HSIL). A total of 331 from 393 swab samples were suitable for detection of HPV infection. Among these, 34.1% (113/331) were positive. HPV 58 (15.9%) was the most common genotype, followed by HPV 18 (14.2%) and HPV 16 (11.5%). The severity of abnormal cells was significantly associated with HPV infection. Dual staining with p16/Ki-67 was performed on 130 samples: in 30.8% (40/130) of samples positive staining was significantly associated with severity of abnormal cells. Agreement between cytology, p16/Ki67 dual-staining and high-risk HPV detection was 100% in HSIL samples. Interestingly, eight apparently NIML cases might have contained abnormal cells, since they were positive by both p16/Ki67 dual-staining and high-risk HPV detection.

**Conclusion:**

Anal specimens screened using modified LBC with 95% ethyl alcohol solution as the fixative are suitable for screening anal precancerous lesions by cytology, HPV testing and p16/Ki-67 dual staining.

## Introduction

Anal cancer has striking similarities with cervical cancer in terms of etiology and pathogenesis. Both are associated with persistent infection by human papillomavirus (HPV) (Albuquerque and Medeiros, 2019). Immunosuppressed state with decreased numbers of CD4+ cells, HIV infection and sexual practices, such as receptive anal intercourse and high lifetime number of sexual partners, are all risk factors for anal HPV infection leading to anal cancer (Kelly et al., 2020). Over the past decade, the incidence rate of anal cancer has increased, particularly in HIV-infected individuals among whom incidence is about 30-fold higher than in the general population (Weis et al., 2011). The weakening immune system of HIV-infected individuals is an additional risk factor for HPV infection that leads to anal intraepithelial neoplasia (AIN) and anal cancer (van der Zee et al., 2013). Screening for cervical cancer by cytology is widely accepted as the best strategy to reduce incidence and mortality rate: anal cytology can perhaps do the same for anal cancer (Darragh and Winkler, 2011). Anal cytology screening is recommended for all at-risk populations, including HIV-infected individuals (Anthony et al., 2015). Anal cytology for detection of AIN is moderately sensitive (60-70%) and specific (32-59%). Additional tests need to be developed cytological technique for increase sensitive and specific to improve these results (Walts et al., 2014). Testing for HPV DNA is an adjunct to cytology, or can be used alone to screen for anal cancer: it has greater sensitivity (75-100%) than cytology (Darragh and Winkler, 2011). However, HPV is very common in the general population and need not be associated with anal cancer. Hence this test is not very specific. In recent years, several biomarkers have been proposed for immunocytochemistry to detect expression of cellular products affected by HPV infection. Observed deregulation of the cell cycle identifies those infections at risk of progression toward dysplasia and carcinoma. Such tests should be more specific than HPV DNA testing. One of the most widely studied markers is the cyclin-dependent kinase inhibitor p16 INK4a (p16) (Tay et al., 2017). Recently, a kit has been developed by CINtec^®^ PLUS Cytology to stain for both p16 and Ki-67. Co-expression of *p16* and *Ki-67* in the same anal cells is an indicator of a deregulated cell cycle caused by HPV oncoproteins and is typical of precancerous cells (Donà et al., 2012). There is limited information on the prevalence of anal HPV on the best strategy of screening for anal cancer. In this study, we investigated the correlation between results from cytology, HPV DNA and p16/Ki-67 dual staining in HIV-infected patients.

## Materials and Methods

*Specimen collection and preparation*

The study protocol was reviewed and approved by the Ethics Committee of Khon Kaen University, Thailand (HE 551148). This study was a cross-sectional analysis of 393 HIV-infected individuals at Srinagarind Hospital and Sirindhorn Hospital in Thailand. Included were all such patients who underwent anal cancer screening between January 2014 and February 2016. Exclusion criteria were; presence of gastrointestinal hemorrhage, intestinal obstruction or anal wound. Anal specimens were collected with Rayon swabs that were gently inserted approximately 5-6 cm into the anal canal while rotating the swab to contact all sides of the anal canal. The anal swab was quickly placed in 95% ethyl alcohol prior to modified-liquid based cytology (Patarapadungkit et al., 2012).

*Anal cytology*

Anal cytology specimens in 95% ethyl alcohol were centrifuged at 1500 rpm for 10 minutes at room temperature. The sediment (approximately 200 µl) was transferred to positively charged slides. The slides were air-dried at room temperature then fixed in 95% alcohol for at least 15 min, and Papanicolaou stains were used to assess cellularity and morphology. All slides were evaluated independently by 2 cytologists. The cytology results were categorized following the Bethesda system (2014) as unsatisfactory specimen contains nucleated squamous cells less than 1-2 per high power field, many anucleated squamous epithelial cells, obscuring bacterial, fecal material and cellular debris, negative for intraepithelial lesion or malignancy (NILM), atypical squamous cells of undetermined significance (ASCUS), low-grade squamous intraepithelial lesion (LSIL), atypical squamous cells cannot rule out high-grade squamous intraepithelial lesion (ASC-H), High-grade squamous intraepithelial lesion (HSIL), and squamous cells carcinoma (SCC) (Nayar and Wilbur, 2015).

*HPV DNA extraction and genotyping*

Anal cells were washed with phosphate buffered saline by centrifugation at 13,500 rpm, 4°C for 5 minutes and DNA was extracted using the AllPrep DNA mini kit (Qiagen, Valencia, USA) according to the manufacturer instruction. Quality of the extracted DNA was evaluated by amplification of the ß-globin gene as an internal control. Samples positive by PCR for the ß-globin gene were subjected to HPV DNA detection by real-time PCR on a LightCycler 480 instrument (Roche Diagnostics). A 142-bp fragment from the L1 region of HPV DNA was amplified using the consensus primers GP5+/GP6+ (van den Brule et al., 2002). The HPV DNA positive samples were used for HPV DNA genotyping by reverse line blot hybridization (RLBH), assaying 37 HPV types including HPV 6, 11,16, 18, 26, 31, 33, 34, 35, 39, 40, 42, 43, 44, 45b, 51, 52, 53, 54, 55, 56, 57, 58, 59, 61, 66, 68, 70, 71 CP8106, 72, 73, 81 CP3804, 82 MM4, 82 IS39, 83 MM7, 84 MM8, CP 6108.

*Immunocytochemistry*

The residual specimens were centrifuged at 1,500 rpm for 10 minutes at room temperature, the sediment was aspirated approximately 200 µl and then transferred to positively-charged slides. The slides were air-dried at room temperature after that fixed in 95% alcohol for at least 15 minutes and dried the slide on a flat horizontal surface for 60 min at room temperature, before performing p16/Ki-67 dual stain. The p16/Ki-67 dual stain study was performed automatically, using VENTANA BenchMark XT equipment and CINtec^®^ PLUS Cytology Kit reagent. The kit designed were used a primary monoclonal mouse antibody directed to human p16 protein and a primary monoclonal rabbit antibody directed against Ki-67 protein. After counterstaining, a two-step mounting protocol must be applied: in the first step an aqueous mounting of the specimens using an aqueous mounting medium. Subsequently, the slides may be cover-slipped using a permanent mounting medium. The result can be evaluated by light microscopy inspection. The positive result of p16/Ki-67 was defined as one or more of anal epithelial cells stained with both a brown cytoplasmic stain (p16) and red nuclear (Ki-67) irrespective of interpretation of morphological abnormalities slides without any double-stained cells were called negative for p16/Ki-67 dual stain.

*Statistical analysis*

Statistical analysis was carried out using SPSS program, version 19.0 for Windows (SPSS, INc., Chicago, IL, USA). The chi-square test was used to quantify any correlation between anal cytology and HPV DNA testing. A value of p <0.05 was considered statistically significant. 

## Results

*Cytological diagnosis*

**Figure 1:**
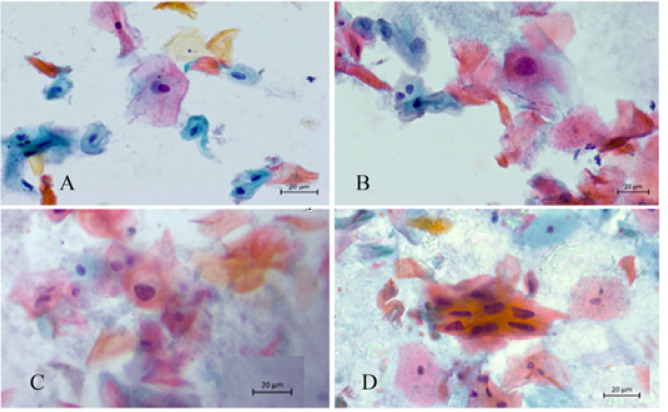
Representative Examples of modified LBC Pap Smear Showing: a. NILM, b. ASCUS, c. LSIL with HPV Infection with kiolocytes; and d. HSIL (arrow) from Papanicolaou stain (x400).

**Figure 2:**
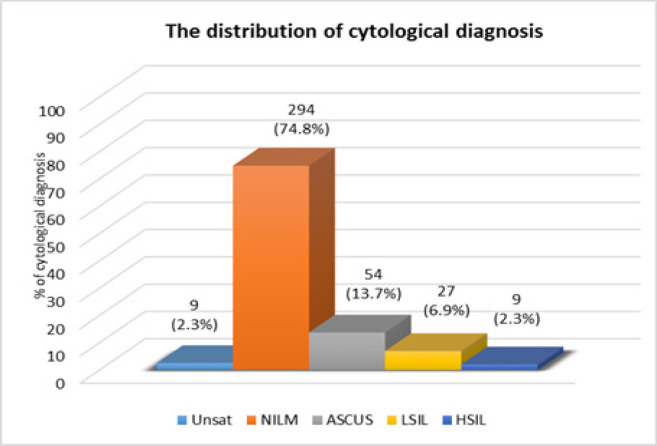
The Distribution of Cytological Diagnosis from HIV-Infected Patients 393 Cases

In total, 393 anal specimens were collected including 64.6% (254/393) from males and 35.4% (139/393) from females. Nine of 393 specimens (2.3%) were regarded as unsatisfactory in modified LBC: fewer than 6 nucleated squamous cells were present per high-power field. Among remaining samples, 74.8% (294/393) were classified as negative for intraepithelial lesion or malignancy (NILM) and 22.9% (90/393) as atypical squamous cells of undetermined significance (ASCUS) following the Bethesda system 2014. The most common categories within abnormal squamous intraepithelial lesion (ASIL) were ASCUS (13.7%; 54/393) followed by low-grade squamous intraepithelial lesion (LSIL) (6.9%; 27/393) and high-grade squamous intraepithelial lesion (HSIL) (2.3%; 9/393) (Figures 1, 2).

*The prevalence of HPV DNA*

Of the 393 anal specimens, valid HPV DNA results were obtained from 331: 62 samples lacking adequate material for DNA extraction were excluded. HPV DNA was not detected in 65.9% (248/331) of samples: 34.1% (113/331) were positive. The most common high-risk HPV genotypes in single-genotype infections were HPV 58 (15.9%; 18/113), HPV 18 (14.2%; 16/113) and HPV 16 (11.5%; 13/113). Other HPV high-risk genotypes present as single infections were HPV 33 (1.8%; 2/113), HPV 45 (0.9%; 1/113), HPV 53 (0.9%; 1/113), HPV 56 (0.9%; 1/113), HPV 70 (0.9%; 1/113) and HPV 73 (0.9%; 1/113). Low-risk HPV genotypes detected were HPV 11 (6.2%; 7/113), HPV 6 (4.4 %; 5/113), HPV 42 (2.7%; 3/113), HPV 72 (1.8%; 2/113) and HPV 43 (0.9%; 1/113). The top three multiple-genotype HPV infections were HPV 11 & 52 (5.3%; 6/113), HPV 6 & 16 (2.7%; 3/113), HPV 11 & 16 (1.8%; 2/113) and HPV 18 & 58 (1.8%; 2/113). HPV DNA was found in 55.8% (63/113) of specimens classed as NILM and in 44.2% (50/113) of those classed as ASIL. In the latter category, HPV DNA was found in 22.1% (25/113) of ASCUS samples, 15.9% (18/113) of LSIL samples and 6.2% (7/113) of HSIL samples (Table 1). The proportions of high-risk HPV infections did not differ significantly between samples classed as ASCUS and those classed as LSIL (p = 0.557). However, a significant difference in prevalence of high-risk HPV was found between samples classed as HSIL which will progress to cervical cancer (p = 0.030).

*p16/Ki-67 dual stain testing in cytological categories*

**Figure 3:**
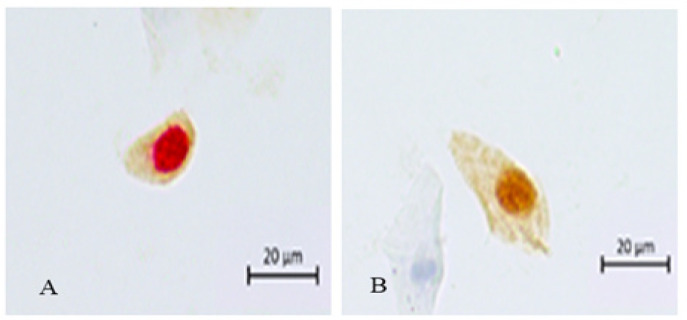
The p16/Ki-67 Dual Stain. HSIL cytology showed. a, Positive with red (Ki-67) staining nuclei and brown (p16) cytoplasm; b, Negative with brown (p16) both of nuclear and cytoplasm but no red (Ki-67) nuclear staining or no staining both of nuclear and cytoplasm

Anal epithelial cells positive for Ki-67 showed red-stained nuclei while those positive for p16 had brown staining in the cytoplasm. The negative results of p16/Ki-67 dual stain were not stained or both of brown staining in the nuclear and cytoplasm of p16 (Figure 3). A total of 130 anal samples were detected by p16/Ki-67 dual stain testing. The 52 negative HPV results were found 100% (32/32) in NILM, but were found positive p16/Ki-67in 18.75% (3/16) of ASCUS and 75% (3/4) of LSIL. All HSIL samples positive for high-risk HPV and Undetermined were also positive for p16/Ki-67. Corresponding values for LSIL, ASCUS and NILM samples were 57.1%, 36.4% and 29.6%, respectively. Low-risk HPV samples were all negative p16/Ki-67 in NILM, but were stained positive for 28.6% (2/7) ASIL; 100% (5/5) LSIL and 50% (1/2) HSIL. The undetermined HPV were positive p16/Ki-67 in both NILM and ASIL which were stained in 100% of LSIL and HSIL (Table 2). 

## Discussion

Screening for anal cancer is recommended for all HIV-infected patients, although no standard guidelines exist. Following the cervical cancer screening model, anal cytology has been proposed for anal cancer screening (Ehrenpreis and Smith, 2018). In this regard, the conventional smear is often air-dry artifact. Liquid-based cytology preparations such as ThinPrep, Liqui-PREP and modified liquid-base cytology yield low background smears due to reduction in fecal material and bacteria, facilitating easier visualization of abnormal cells (Patarapadungkit et al., 2012; Pankam et al., 2017; Khattab et al., 2018). We found abnormal anal cytology in 22.9% of HIV-infected patients. Another study in Thailand reported the prevalence of ASIL among HIV-infected men who have sex with men (MSM) to be 43.0% (Ruanpeng et al., 2016). Among samples in the ASIL category, 13.7% were in the ASCUS subcategory, a lower proportion than reported in another study on HIV-infected MSM (between 16.7% and 25.7%) (Weis et al., 2011). We classified 2.3% of samples as HSIL, which is considered the real precursor of anal carcinoma. LSIL, at 6.9%, was a more frequent diagnosis than HSIL. It is worth noting that LSIL might be clinically important. In one study, about 62% of HIV-positive MSM and 36% of HIV-negative MSM who had a diagnosis of LSIL at baseline progressed to HSIL within 2-4 years (Palefsky et al., 1998). A diagnosis of ASCUS may also hide squamous intraepithelial lesions (Wang et al., 2019). The presence of any abnormal anal cytological finding indicates a potential association with high-grade anal intraepithelial neoplasia (AIN) on pathological examination (Machalek et al., 2016). These data underline that all patients with a cytological report of ASCUS or worse need a follow-up for accurate diagnostic investigation. Importantly, HPV is the most common sexually transmitted viral disease implicated in progression of anal intraepithelial lesion into squamous cell carcinoma. HPV has a particularly high prevalence among HIV-infected patients. HPV DNA testing is recommended an alternative or an adjunct to cytology (Dias et al., 2019). In our anal samples collected for modified LBC, the prevalence of HPV infection was 34.14%, which lies within the range noted in previous publications (Gandra et al., 2015; Stier et al., 2015). It should be noted that the prevalence of ASIL and HPV infection varies according to sexual behavior and geography. Furthermore, infection by any HPV is associated with a 4-fold increased risk of having abnormal anal cytology (Donà, et al., 2012). In our study, the prevalence of HPV infection seems to rise with grade of cytological abnormality. We found HPV infection, both with single and multiple genotypes, in all ASIL samples. Infections with two or more HPV genotypes is common in anal specimens (Dietrich et al., 2015). The prevalence of high-risk HPV single infection was 47.8%. Corresponding values for women and for HIV-infected men who have sex with men range from 37.5% (Fan et al., 2020). Among high-risk HPV types, we found HPV 58 to be the most common, followed by HPV 18, HPV 16 and HPV 33. The most common high-risk HPV types in HIV patients worldwide are HPV 16 followed by HPV 18 (Fan et al., 2020; Nasioutziki et al., 2020). Among Spanish MSM, the most prevalent high-risk HPV was HPV 31 and in China, HPV 58 were the most frequently detected (Torres et al., 2013; Liu et al., 2019). As for low-risk HPV types, HPV 11, HPV 6 and HPV 42 were the most frequently detected. Abnormal anal cytology can be associated with low-risk HPV types HPV6, HPV 11, HPV 40 and HPV 42 (Zhang et al., 2014). The frequency of HPV genotypes varies by geographic region. The high-risk HPV infections were detected in cases with each cytological diagnosis including negative, ASCUS, LSIL and HSIL. However, it should be noted that HPV DNA testing has high sensitivity but low specificity as a screening tool for cancer (Kang et al., 2020). The presence of high-risk HPV infection in cases classed as ASCUS may reflect the fact that a diagnosis of ASCUS is consistent with benign reactive changes other than malignancy. Similarly, in cases classed as LSIL by cytology, we found infection with high-risk HPV. LSIL may be a consequence of low-risk HPV infection and other causes. HSIL cases were positive for HPV DNA including high-risk HPV, low-risk HPV and multiple infections with both. We found HSIL to be strongly associated with high-risk HPV infection, as also reported by others (Dietrich et al., 2015). In the immunocytochemistry study, we conducted p16/Ki-67 dual staining on a selected series of anal specimens with all grades of abnormal cytology and/or HPV DNA-positive. We investigated the correlation between p16/Ki-67 dual staining and the cytological diagnosis and high-risk HPV infection. There was a significant increase in samples positive for p16/Ki-67 with increasing severity of the cytological lesion, especially in HISL with high-risk HPV infection. Nevertheless, we found 8 cases (29.63%) of cytologically normal samples with high-risk HPV infection and positive for p16/Ki-67 staining. It may be that these 8 cases are also precancerous despite their normal appearance. Similarly, Lee and Lee (2016) demonstrated that 5 of 6 cervical samples of ASCUS and LSIL with p16 methylation progressed to HSIL (Lee and Lee, 2016). It is worth noting that 8 of 17 cases (47.06 %) of ASIL that were positive for low-risk HPV were also positive for p16/Ki-67. One follow-up study on cervical cases classified as ASCUS and negative for high-risk HPV found that, one year later, 23% had progressed to LSIL and 4% to HSIL (Ozturk et al., 2016). Furthermore, Balmagambetova et al., (2019), in a review of the literature, noted the reported presence of low-risk HPV types 6 and 11 in invasive anal cancer cases (Balmagambetova et al., 2019) and Serrano et al., (2015) reported low-risk HPV associated with cancers of the anus, cervix, vagina and vulva (Serrano et al., 2015). Several previous studies have reported p16/Ki-67 positivity is independent of HPV genotypes and achieved a sensitivity equal to HPV testing with significantly higher specificity (Zhang et al., 2019; Hu et al., 2020). Consequently, the presence of p16/Ki-67 staining in normal cells or dysplastic cells should not be ignored and may indicate deregulation of the cell cycle. In this study, we detected p16/Ki-67 dual stain in anal specimens positive for HPV or cytological diagnosis as ASIL including ASCUS, LSIL and HSIL. Such staining was not detected in NILM samples positive for low-risk HPV or without HPV. Therefore, the screening of anal samples with p16/Ki-67 dual stain should be done when the patient has a cytological diagnosis of ASCUS or LSIL, with or without HPV infection. Limitations of our study were that a small number of cases were analyzed by p16/Ki-67 dual staining and no biopsy was taken to confirm specificity. 

In conclusion, anal specimens screened using modified LBC with 95% ethyl alcohol solution as the fixative are suitable for screening anal precancerous lesions by cytology, HPV testing and p16/Ki-67 dual staining. This study should contribute to management guidelines for anal cancer screening. If the results of cytology were ASCUS and LSIL, with or without the presence of HPV DNA, further investigation by p16/Ki-67 dual staining is worthwhile.

**Table 1:** The Prevalence of HPV Detection and Genotyping in Cytological Diagnosis 113 Cases

HPV genotypes	Cytological diagnosis				Total n (%)
	NILM	ASCUS	LSIL	HSIL	
	n (%)	n (%)	n (%)	n (%)	
Single infection (any type)	41/63 (65.1)	16/25 (64.0)	11/18 (61.1)	4/7 (57.1)	72/113 (63.7)
Any high-risk type	37/63 (58.7)	9/25 (36.0)	6/18 (33.3)	2/7 (28.6)	54/113 (47.8)
HPV 16	8 (12.7)	2 (8.0)	2 (11.1)	1 (14.3)	13 (11.5)
HPV 18	9 (14.3)	5 (20.0)	2 (11.1)	-	16 (14.2)
HPV 33	1 (1.6)	-	1 (5.6)	-	2 (1.8)
HPV 45	1 (1.6)	-	-	-	1 (0.9)
HPV 53	1 (1.59)	-	-	-	1 (0.9)
HPV 56	-	-	-	1 (14.3)	1 (0.9)
HPV 58	16 (25.4)	1 (4.0)	1 (5.6)	-	18 (15.9)
HPV 70	1 (1.6)	-	-	-	1 (0.9)
HPV 73	-	1 (4.00)	-	-	1 (0.9)
Any low-risk type	4/63 (6.4)	7/25 (28.0)	5/18 (27.8)	2/7 (28.6)	18/113 (15.9)
HPV 6	1 (1.6)	3 (12.0)	1 (5.6)	-	5 (4.4)
HPV 11	2 (3.2)	2 (8.0)	2 (11.1)	1 (14.3)	7 (6.2)
HPV 42	1 (1.6)	1 (4.0)	-	1 (14.3)	3 (2.7)
HPV 43	-	-	1 (5.6)	-	1 (0.9)
HPV 72	-	1 (4.0)	1 (5.6)	-	2 (1.8)
Multiple infection	11/63 (17.5)	6/25 (24.0)	5/18 (27.8)	3/7 (42.9)	25/113 (22.2)
HPV 6,16	-	1 (4.0)	-	2 (28.6)	3 (2.7)
HPV 6,54	1 (1.6)	-	-	-	1 (0.9)
HPV 11,16	2 (3.2)	-	-	-	2 (1.8)
HPV 11,45	-	-	1 (5.6)	-	1 (0.9)
HPV 11,52	3 (4.8)	1 (4.0)	2 (11.1)	-	6 (5.3)
HPV 11,70	-	-	1 (5.6)	-	1 (0.9)
HPV 16,56	-	-	1 (5.6)	-	1 (0.9)
HPV 16,70	-	1 (4.0)	-	-	1 (0.9)
HPV 16,72	-	1 (4.0)	-	-	1 (0.9)
HPV 18,53	1 (1.6)	-	-	-	1 (0.9)
HPV 18,56	-	1 (4.0)	-	-	1 (0.9)
HPV 18,58	2 (3.17)	-	-	-	2 (1.8))
HPV 33,43	-	-	-	1 (14.3)	1 (0.9)
HPV 44,56	-	1 (4.0)	-	-	1 (0.9)
HPV 52,58	1 (1.6)	-	-	-	1 (0.9)
HPV 56,58	1 (1.6)	-	-	-	1 (0.9)
Undetermined	11/63 (17.5)	3/25 (12.0)	2/18 (11.1)	-	16/113 (14.2)
Total	63 (55.8)	25 (22.1)	18 (15.9)	7 (6.2)	113

**Table 2:** The Positive of p16/Ki-67 in Anal Cytology Categories

HPV status	P16/Ki67	NILM	ASCUS	LSIL	HSIL	Total
HPV Negative (52)	negative (%)	32 (100)	13 (81.3)	1 (25.0)	0 (0.0)	46 (88.5)
	positive (%)	0 (0.0)	3 (18.7)	3 (75.0)	0 (0.0)	6 (11.5)
HR HPV positive (49)	negative (%)	19 (70.4)	7 (63.6)	3 (42.9)	0 (0.0)	29 (59.2)
	positive (%)	8 (29.6)	4 (36.4)	4 (57.1)	4 (100.0)	20 (40.8)
LR HPV positive (17)	negative (%)	3 (100.0)	5 (71.4)	0 (0.0)	1 (50.0)	9 (52.9)
	positive (%)	0 (0.0)	2 (28.6)	5 (100.0)	1 (50.0)	8 (47.1)
Undetermined (12)	negative (%)	5 (71.4)	1 (50.0)	0 (0.0)	0 (0.0)	6 (50.0)
	positive (%)	2 (28.6)	1 (50.0)	2 (100.0)	1 (100.0)	6 (50.0)
Total		(53.1) 69	(27.7) 36	(13.8) 18	(5.4) 7	130
